# Phytochemical Profiling, Antioxidant and Antimicrobial Potentials of Ethanol and Ethyl Acetate Extracts of *Chamaenerion latifolium* L.

**DOI:** 10.3390/ph17080996

**Published:** 2024-07-27

**Authors:** Akmaral Kozhantayeva, Nurgul Tursynova, Ainagul Kolpek, Yelaman Aibuldinov, Arailym Tursynova, Togzhan Mashan, Zhazira Mukazhanova, Manshuk Ibrayeva, Aizhan Zeinuldina, Aisha Nurlybayeva, Zhanar Iskakova, Yerbolat Tashenov

**Affiliations:** 1 Research Institute of New Chemical Technologies, L.N. Gumilyov Eurasian National University, Satpayev Street 2, Astana 010000, Kazakhstan; akmaral-muslim@mail.ru (A.K.); n_him@mail.ru (N.T.); elaman_@mail.ru (Y.A.); 2 Department of Chemistry, Faculty of Natural Sciences, L.N. Gumilyov Eurasian National University, Satpayev Street 2, Astana 010000, Kazakhstan; aynagulk@mail.ru (A.K.); tursynova_79@mail.ru (A.T.); togzhan-mashan@mail.ru (T.M.); 3 Department of Chemistry, Graduate School of IT and Natural Sciences, East Kazakhstan University Named after S. Amanzholov, Ust-Kamenogorsk 010008, Kazakhstan; mukazhanovazhb@mail.ru; 4 Faculty of Science and Technology, The Caspian University of Technology and Engineering Named after Sh.Yessenov, Aktau 130000, Kazakhstan; ibrayevamanshuk@mail.ru; 5 Department of General and Biological Chemistry, NJSC “Astana Medical University”, Astana 010000, Kazakhstan; zeinuldina.a@amu.kz; 6 Department of Chemistry and Chemical Technology, Faculty of Technology, M.Kh. Dulaty Taraz Regional University, Taraz 080000, Kazakhstan; rustem_ergali@mail.ru

**Keywords:** *Chamaenerion latifolium* L., HPLC-UV-ESI/MS analysis, FT-IR profile, antioxidant activity, antimicrobials

## Abstract

The study investigates the phytochemical profile, antioxidant capacity, and antimicrobial activities of ethanol (ChL-EtOH) and ethyl acetate (ChL-EtOAc) extracts from *Chamaenerion latifolium* L. (ChL) harvested in Kazakhstan. The ChL-EtOH extract exhibited higher total phenolic (267.48 ± 3.44 mg GAE/g DE) and flavonoid content (24.18 ± 1.06 mg QE/g DE) compared to ChL-EtOAc. HPLC-UV-ESI/MS identified key phenolic acids and flavonoids, including gallic acid, chlorogenic acid, and quercetin 3-glucoside. FT-IR analysis confirmed the presence of characteristic functional groups. Antioxidant assays revealed strong DPPH scavenging and FRAP activities, with ChL-EtOH showing superior results (IC_50_ = 21.31 ± 0.65 μg/mL and 18.13 ± 0.15 μg/mL, respectively). Additionally, ChL-EtOH displayed notable antimicrobial efficacy against Gram-positive and Gram-negative bacteria, as well as the fungal strain *Candida albicans*. These findings suggest that ethanol extraction is more efficient for isolating bioactive compounds from ChL, underscoring its potential for pharmaceutical and nutraceutical applications.

## 1. Introduction

The search for natural bioactive compounds has gained significant momentum in recent years due to growing concerns over the side effects associated with synthetic drugs and increasing resistance to antibiotics [1,2,3]. Medicinal plants, with their rich repository of bioactive compounds, have been a cornerstone of traditional medicine for centuries and are now being rigorously investigated for their potential applications in modern pharmaceuticals and nutraceuticals and various other fields, including dentistry [4,5,6]. *Chamaenerion latifolium* L. (ChL), commonly known as dwarf fireweed or river beauty, is one such plant that has garnered attention for its purported medicinal properties. Belonging to the *Onagraceae* family, this perennial herbaceous plant is native to temperate regions, including parts of North America, Europe, and Asia, particularly in the Almaty region of Kazakhstan [7,8].

Traditionally, the *Chamaenerion* species has been used in various cultures for its anti-inflammatory, antioxidant, and antimicrobial properties [9,10,11,12]. These therapeutic benefits are primarily attributed to its rich content of phenolic compounds and flavonoids [13,14]. Phenolic compounds, including phenolic acids and flavonoids, are well-known for their strong antioxidant activities, which can mitigate oxidative stress and reduce the risk of chronic diseases such as cancer, cardiovascular diseases, and neurodegenerative disorders [15,16,17]. Furthermore, flavonoids possess a wide range of biological activities, including anti-inflammatory, antibacterial, antiviral, and anticancer properties, making them valuable compounds in both preventive and therapeutic medicine [18,19,20].

The efficacy of plant-based extracts largely depends on the extraction methods and solvents used. Solvent extraction is a critical step in the isolation of bioactive compounds from plant materials [21]. The choice of solvent can significantly influence the yield and composition of the extracted compounds [22,23]. Ethanol and ethyl acetate are commonly used solvents in phytochemical studies due to their ability to dissolve a wide range of bioactive substances [24,25,26,27]. Chosen for their different polarities, ethanol and ethyl acetate allow comprehensive extraction of both polar and non-polar compounds. Ethanol is particularly effective in extracting phenolic compounds due to its polar nature and ability to penetrate plant tissues efficiently, while ethyl acetate targets less polar substances, ensuring a diverse phytochemical profile from ChL [28,29].

According to the traditional use and potential therapeutic benefits of *Chamaenerion* species, it seems of interest to investigate the chemical composition and biological activities of ChL extracts, which have not been studied to date. Most research has focused on related species such as *C. angustifolium* L., also known as fireweed or willowherb, which has been studied for its phenolic content and associated health benefits [30,31,32]. Studies on *C. angustifolium* have identified several phenolic acids, including caffeic acid, gallic acid, and chlorogenic acid, and flavonoids such as quercetin, myricetin, and kaempferol, which exhibit significant antioxidant and antimicrobial activities [33,34,35,36]. Given the pharmacological potential of these compounds, it is crucial to investigate the phytochemical composition and biological activities of ChL to explore its potential applications in modern medicine. This study aims to fill the knowledge gap by evaluating the phytochemical profile, antioxidant capacity, and antimicrobial activities of ethanol and ethyl acetate extracts from ChL harvested in the Almaty region of Kazakhstan. By employing a combination of spectrophotometric assays, high-performance liquid chromatography (HPLC-UV-ESI/MS), and Fourier-transform infrared spectroscopy (FT-IR), this research provides a comprehensive analysis of the extracts’ chemical compositions and bioactivities.

The primary objectives of this study are threefold: (1) to compare the extraction yields and efficiency of ethanol and ethyl acetate solvents in isolating bioactive compounds from ChL; (2) to determine the total phenolic content (TPC) and total flavonoid content (TFC) of the extracts and identify individual phenolic compounds using HPLC-UV-ESI/MS; and (3) to evaluate the antioxidant and antimicrobial activities of the extracts using in vitro assays. By achieving these objectives, this study aims to highlight the potential of ChL as a source of natural antioxidants and antimicrobial agents, providing a scientific basis for its inclusion in pharmaceutical and nutraceutical formulations.

## 2. Results and Discussion

### 2.1. Extraction Yield Analysis

The Soxhlet extraction process utilizing both ethanol and ethyl acetate solvents yielded significant quantities of extract from ChL plant material. The extraction yields for ethanol and ethyl acetate extracts, denoted hereinafter as ChL-EtOH and ChL-EtOAc, were determined as 6.22 ± 0.52 g (31.1 ± 2.6% *w*/*w*) and 3.14 ± 0.26 g (15.7 ± 1.3% *w*/*w*), respectively, based on the initial dry mass of the plant material (*n* = 3). The results indicate that ethanol extraction yielded a higher quantity of extract compared to the extraction with ethyl acetate. The higher extraction yield with ethanol can be attributed to its polar nature, which makes it highly effective at penetrating plant cell walls and extracting a wide range of polar bioactive compounds, including phenolic acids and flavonoids. Ethyl acetate, being less polar, is less efficient at extracting these compounds, resulting in a lower yield.

### 2.2. Phytochemical Study

#### 2.2.1. Total Phenolic and Flavonoid Content

The primary objective of this research was to quantify the major bioactive compounds, specifically phenols and flavonoids, in ChL from the Almaty region of Kazakhstan. The TPC and TFC values of ChL-EtOH and ChL-EtOAc extracts were determined using standard spectrophotometric methods. Preliminary colorimetric tests were followed by a more precise analysis using liquid chromatography with HPLC-UV-ESI/MS for its high specificity and accuracy. Table 1 presents the TPC values determined by the Folin–Ciocalteu method and the TFC results for both extracts. The TFC results showed that the ethanol extract had a slightly higher flavonoid concentration (24.18 ± 1.06 mg QE/g DM) compared to the ethyl acetate extract (21.72 ± 0.54 mg QE/g DM). This finding is consistent with previous studies suggesting the efficacy of ethanol in extracting flavonoids from botanical sources [37]. A significant difference in total phenolic content was observed between the two extracts. The ChL-EtOAc had a TPC of 42.68 ± 2.48 mg GAE/g DM. In contrast, the ChL-EtOH exhibited a substantially higher phenolic content, with a TPC of 267.48 ± 3.44 mg GAE/g DM. This disparity underscores the superior extraction efficiency of ethanol for phenolic compounds, aligning with existing literature highlighting ethanol as a preferred solvent for maximizing phenolic yield [38,39]. Overall, this study underscores the significance of solvent selection in optimizing the extraction of bioactive compounds from ChL. Ethanol emerges as a preferred solvent for maximizing the yield of phenolic and flavonoid compounds, thus offering promising possibilities for further research and industrial applications [40].

#### 2.2.2. HPLC-UV-ESI/MS Analysis

**Figure 1:**
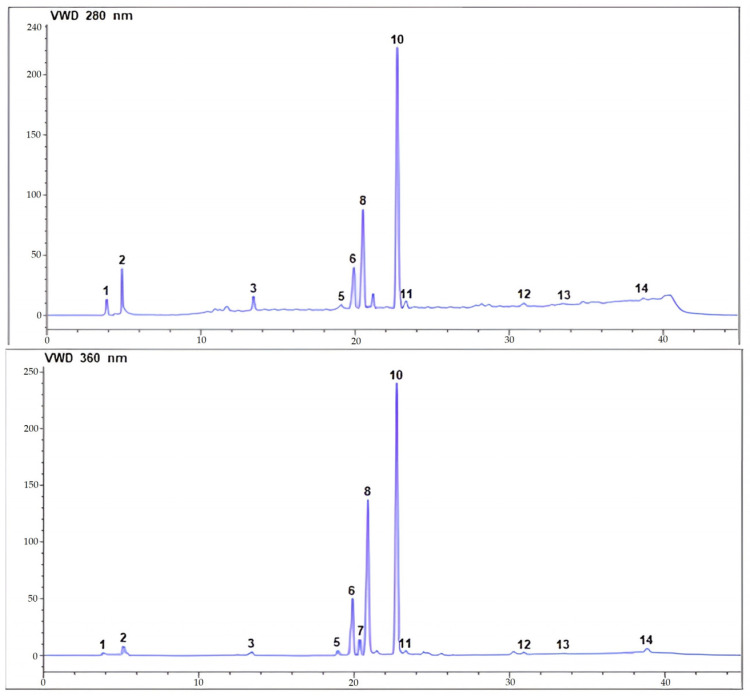
Chromatographic profiles of ethyl acetate extract from ChL acquired at 280 and 360 nm using HPLC-UV-ESI/MS.

**Figure 2:**
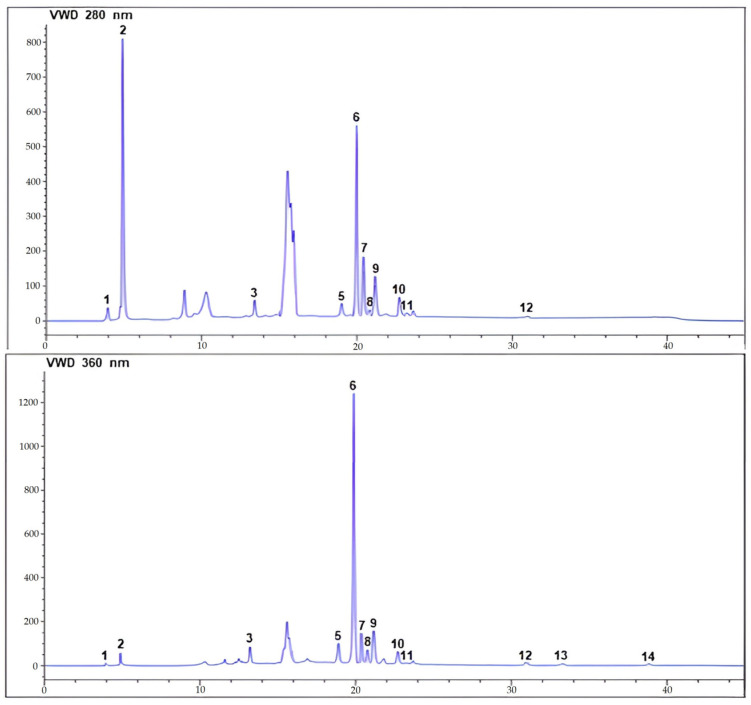
Chromatographic profiles of ethanol extract from ChL acquired at 280 and 360 nm using HPLC-UV-ESI/MS.

The combined analysis using HPLC-UV and ESI-MS/MS offered a comprehensive insight into the chemical compositions of both ethanol and ethyl acetate extracts from ChL. These findings hold particular significance in relation to the therapeutic activities investigated in this study. To identify each compound, we compared their HPLC-UV retention times and HPLC-ESI-MS/MS mass spectra with data from the chromatograms of the authentic phenolic standards. Phenolic compounds were quantified by integrating peaks on HPLC-UV chromatograms using an external standard method. Chromatographic analysis of both extracts at 280 and 360 nm revealed the presence of phenolic acids and flavonoids. The HPLC-UV-ESI/MS analysis of the aerial parts of ChL led to the detection of 13 compounds, with 10 positively identified based on retention times, maximum absorption wavelengths (λ_max_), mass spectrometry data, and existing literature (Figure 1 and Figure 2, Table 2). Three compounds remained unidentified, and no peak for epicatechin was observed in either extract. Each compound was labeled according to its elution order.

**Figure 3:**
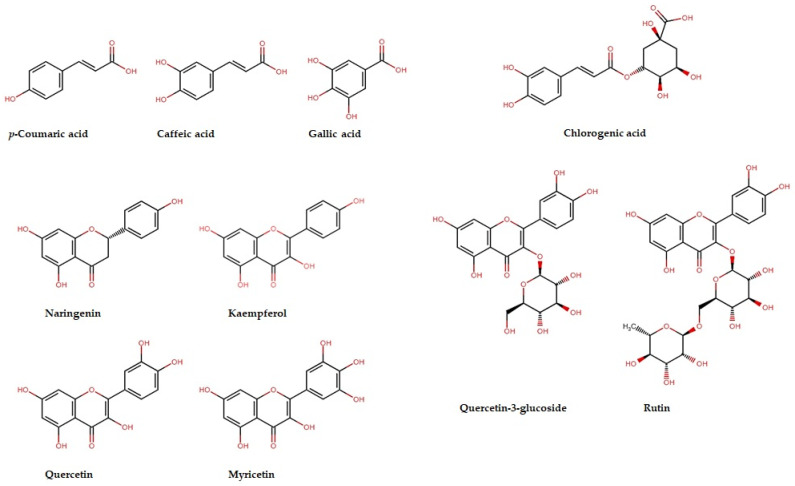
The chemical structures of secondary metabolites identified in ChL extracts.

The chromatographic analysis revealed the presence of several phenolic acids, flavonoids, and flavonoid glycosides in both the ChL-EtOH and ChL-EtOAc extracts. Specifically, caffeic acid, gallic acid, chlorogenic acid, p-coumaric acid, myricetin, quercetin, naringenin, kaempferol, rutin, and quercetin 3-glucoside were identified and quantified in the studied extracts (Figure 3). Quantitative analysis indicated variations in the concentrations of these bioactive compounds between the ethanol and ethyl acetate extracts. For instance, in the ChL-EtOH extract, the highest concentrations were observed for gallic acid (35.00 mg/g), followed by chlorogenic acid (26.80 mg/g), whereas the ChL-EtOAc extract exhibited relatively lower concentrations of these compounds. Similarly, quercetin 3-glucoside was predominantly present in the ChL-EtOH extract (71.90 mg/g) compared to the ChL-EtOAc extract (2.30 mg/g).

As far as we are aware, this is the first-ever examination of the phenolic compound composition within ChL extracts. Our findings corroborate with previous studies on phytochemical analysis of *Chamaenerion* species, albeit with some variations in compound concentrations. For instance, a study by Nowak et al. conducted on *C. angustifolium* reported the presence of caffeic acid, gallic acid, and chlorogenic acid in ethanol extracts [41]. Additionally, research by Maruska et al. on *C. angustifolium* identified quercetin, myricetin, and kaempferol in hydromethanolic extracts [36]. It is important to note that the presence and concentrations of these compounds in plants are greatly affected by the location and season of collection, extraction techniques, and solvent systems used. These variables can result in variations in the content of these compounds among related plant species [42,43,44].

The presence of phenolic compounds in ChL extracts suggests their potential medicinal value, particularly in terms of antioxidant and antimicrobial activities. Caffeic acid, gallic acid, and chlorogenic acid have been widely recognized for their antioxidant properties, which could contribute to the overall therapeutic efficacy of the extracts [45,46,47]. Similarly, flavonoids such as quercetin and kaempferol exhibit significant antioxidant and antimicrobial effects, suggesting their role in enhancing the medicinal potential of ChL extracts [18,48,49,50,51]. Further research into the specific mechanisms of action and bioavailability of these compounds is warranted to fully exploit their medicinal benefits. Additionally, studies evaluating the synergistic effects of these compounds in combination with other phytochemicals present in ChL extracts would provide valuable insights into their therapeutic applications.

#### 2.2.3. FT-IR Analysis

**Figure 4:**
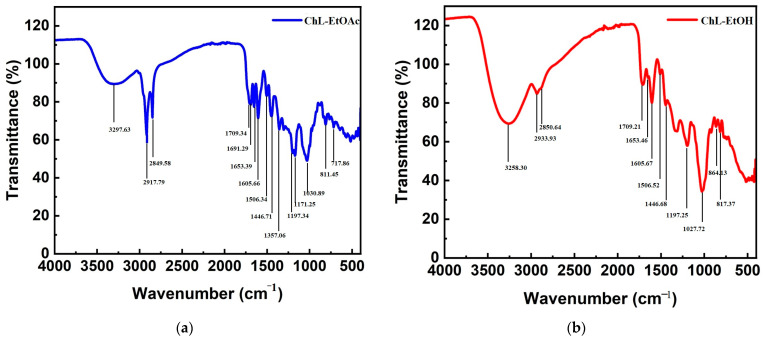
FT-IR spectra of (**a**) ethyl acetate and (**b**) ethanol extracts of ChL.

To supplement the polyphenolic profile, the FT-IR technique, commonly employed to elucidate the characteristic fingerprints of functional groups in secondary metabolites found in plant extracts [37,52,53], was utilized to investigate extracts of ChL. The FT-IR spectra of ChL crude extracts obtained using ethyl acetate and ethanol solvents are illustrated in Figure 4, revealing distinct signals corresponding to individual molecules at specific wavenumbers.

The FT-IR analysis outcomes for both ethyl acetate and ethanol extracts of ChL are presented in Table S1.

The FT-IR spectra of ChL extracts, obtained using ethyl acetate and ethanol solvents, exhibit diverse functional groups, each characterized by specific peak values. In both extracts, the prominent, broad, and well-defined peaks observed at 3297.63 and 3258.30 cm^−1^ suggest the presence of stretching vibrations associated with O-H bonds, commonly found in hydroxyl groups of alcohols and phenols [54]. Additionally, the double bonds at 2917.79/2849.58 cm^−1^ and 2933.93/2850.64 cm^−1^ are attributed to C-H stretching vibrations in aliphatic hydrocarbons, indicative of methyl and methylene groups [55]. These peaks suggest the attachment of aromatic rings and alkyl groups to the C-H stretching functional group or indicate the presence of O-H functional groups originating from chlorogenic acid and flavonoid glycosides, such as rutin and quercetin-3-glucoside.

Moreover, bands at approximately 1710 cm^−1^ in both ethyl acetate and ethanol extracts correspond to the stretching vibration of the carbonyl group (C=O), potentially indicating the presence of carboxyl groups in phenolic compounds and keto groups in flavonoids [56]. It should be noted that in the FTIR spectrum of the ChL-EtOAc extract, lesser peaks for COOH functionalities were detected, which may suggest a lower concentration of carboxyl-containing compounds in this extract. Furthermore, the peak at 1691.29 cm^−1^ in the ethyl acetate extract is associated with C=O stretching vibrations in conjugated carboxylic acids, such as p-coumaric and caffeic acids. Additionally, a band found at 1653 cm^−1^ could be attributed to the stretching vibration of C=C groups of conjugated alkene systems present in p-coumaric, caffeic, and chlorogenic acids. Likewise, the peaks observed at 1605.66 cm^−1^ and 1506.34/1506.52 cm^−1^ possibly indicate C=C stretching vibrations, affirming the presence of the aromatic ring system in both extracts [57].

Furthermore, bands at 1446.71 cm^−1^ and 1357.06 cm^−1^ suggest the presence of O-H bending vibrations in alcohol and carboxylic acid group-containing compounds. Additionally, the stretching of phenolic C–O bonds was detected around 1200 cm^−1^, attributed to the C–O bonds of pyran, characteristic of flavonoid C-rings. These frequency groups are closely associated with the presence of aromatic compounds. Several aromatic out-of-plane C–H (670–900 cm^−1^) and in-plane (950–1225 cm^−1^) bending bonds, along with additional C=C bending bonds of alkene and C-H bending bonds of alkane, may correspond to residual peaks [58]. Notably, a broad strong peak observed at around 1030 cm^−1^ for both extracts may be associated with sulfoxide functional groups, suggesting the presence of compounds with unique molecular structures, warranting further analysis and identification.

These observations align with the presence of phenolcarboxylic acids (such as caffeic acid, gallic acid, chlorogenic acid, and p-coumaric acid) and flavonoids (including rutin, quercetin-3-glucoside, quercetin, naringenin, kaempferol, and myricetin) identified through HPLC analysis in both extracts. The peaks observed in the FT-IR spectra corroborate the characteristic vibrations of these compounds, confirming their presence in the extracts.

### 2.3. Antioxidant Capacity

The antioxidant potential of extracts from the aerial parts of ChL was assessed using two different in vitro methods: DPPH scavenging activity and the ferric reducing antioxidant power (FRAP) assay. Employing these diverse methods aimed to capture the various antioxidant mechanisms in the plant extract. The results are summarized in Table 3.

The most common method to measure the antioxidant or antiradical activity of plant extracts, individual secondary metabolites, and other therapeutic agents is the 2,2-diphenyl-1-picrylhydrazyl (DPPH) test. The DPPH assay is based on the neutralization of the DPPH radical through the contribution of electrons from antioxidants. In the DPPH assay, both extracts from ChL demonstrated noteworthy radical scavenging activity. The ethanolic extract showed slightly higher DPPH scavenging activity (IC_50_ = 22.60 ± 0.44 μg/mL) compared to the ethyl acetate extract, with an IC_50_ value of 21.31 ± 0.65 μg/mL, close to that of the positive control, butylated hydroxytoluene (BHT, IC_50_ = 20.02 ± 0.48 μg/mL). This suggests that the ChL-EtOH extract has strong radical scavenging abilities.

The FRAP assay evaluates antioxidant power based on the reduction of ferric ion (Fe^3+^) to ferrous ion (Fe^2+^). The ChL-EtOH extract exhibited superior antioxidant power, with an IC_50_ value (18.13 ± 0.15 μg/mL) even lower than that of the positive control, ascorbic acid (AA, IC_50_ = 18.64 ± 0.32 μg/mL), indicating its high reducing capability. The ChL-EtOAc extract also showed considerable antioxidant activity with an IC_50_ of 21.62 ± 0.22 μg/mL, though it was slightly less effective than the ChL-EtOH extract.

As far as we know, there have been no prior studies evaluating the antioxidant activity of ChL extracts, particularly within Kazakhstan. Importantly, our findings highlight the significance of comprehending the complex antioxidant mechanisms displayed by plant extracts. The higher antioxidant activity of the ChL-EtOH extract correlates with its higher TPC and TFC values. The comprehensive chemical analysis using HPLC-UV-ESI/MS further supports these findings. The ChL-EtOH extract contained higher concentrations of several phenolic acids and flavonoids, such as gallic acid, chlorogenic acid, and quercetin 3-glucoside, compared to the ChL-EtOAc extract. These compounds are well-documented for their potent antioxidant activities, which likely contribute to the higher antioxidant potential observed in the ChL-EtOH extract [59,60,61,62,63]. Moreover, endogenous phenolic compounds may work together to boost the overall antioxidant activity of the extract, emphasizing the need for further research into their synergistic effects.

### 2.4. Antibacterial and Antifungal Activities

The antibacterial and antifungal activities of ChL extracts were evaluated against a range of bacterial and fungal strains. The bacterial strains included Gram-negative (*Escherichia coli*, *Klebsiella pneumoniae*) and Gram-positive (*Staphylococcus aureus*, *Bacillus cereus*) bacteria, while *Candida albicans* represented the fungal strain. The disc diffusion method was employed to determine the inhibition zones, providing a measure of antimicrobial activity.

The results revealed significant antimicrobial activity of the ChL-EtOH extract across all tested strains. Specifically, against *E. coli*, *K. pneumoniae*, *S. aureus*, *B. cereus*, and *C. albicans*, the ChL-EtOH extract exhibited inhibition zone diameters (IZD) of 8.53 ± 0.12 mm, 9.26 ± 0.08 mm, 11.06 ± 0.30 mm, 9.60 ± 0.24 mm, and 14.27 ± 0.65 mm, respectively. In contrast, the ChL-EtOAc extract showed no activity against the bacterial strains but displayed activity against *C. albicans* with an IZD of 8.58 ± 0.22 mm (Table 4, Figure S1).

The high antimicrobial activity of the ChL-EtOH extract can be attributed to its rich composition of phenolic acids and flavonoids, as determined by HPLC-UV-ESI/MS analysis. Notably, the ChL-EtOH extract contained higher quantities of caffeic acid (4.40 mg/g), gallic acid (35.00 mg/g), chlorogenic acid (26.80 mg/g), rutin (8.80 mg/g), and quercetin 3-glucoside (71.90 mg/g) compared to the ChL-EtOAc extract. These compounds are well-documented for their potent antimicrobial properties [64,65,66,67]. For instance, chlorogenic acid has been shown to exhibit strong antibacterial and antifungal activities, contributing to the overall efficacy of plant extracts [68,69].

Previous studies have highlighted the importance of phenolic acids and flavonoids in antimicrobial activity [70]. For example, Khan et al. reported significant antibacterial activity of caffeic acid and its derivatives against both Gram-positive and Gram-negative bacteria [64]. Additionally, quercetin and its glycosides, such as quercetin 3-glucoside, have been recognized for their ability to disrupt bacterial cell walls and membranes, leading to increased cellular permeability and inhibition of essential microbial processes [71].

The antimicrobial activity observed in this study aligns with these findings. The higher inhibition zones for the ChL-EtOH extract against Gram-positive bacteria, such as *B. cereus* and *S. aureus*, may be due to the thicker peptidoglycan layer in these bacteria, which is more susceptible to disruption by phenolic compounds. In contrast, the Gram-negative bacteria *E. coli* and *K. pneumoniae*, with their additional outer membrane, exhibited lower sensitivity, a common trend observed in antimicrobial studies of plant extracts [72]. The antifungal activity against *C. albicans* was particularly noteworthy, with the ChL-EtOH extract demonstrating an inhibition zone of 14.27 ± 0.65 mm, surpassing the positive control antibiotic nystatin, which had an IZD value of 11.28 ± 0.28 mm, indicative of its strong antifungal potential. This efficacy can be attributed to the high concentration of phenolic acids and flavonoids, which are known to inhibit fungal growth through various mechanisms, including disruption of cell membrane integrity and interference with fungal enzyme activity [73,74].

In summary, the ChL-EtOH extract of ChL exhibited significant antibacterial and antifungal activities, surpassing the ChL-EtOAc extract. The robust antimicrobial properties of the ethanolic extract can be directly correlated with its higher content of bioactive phenolic compounds and flavonoids, emphasizing the potential of ChL as a source of natural antimicrobial agents. Further research is warranted to explore the synergistic effects of these compounds and to fully elucidate the mechanisms underlying their antimicrobial actions.

## 3. Materials and Methods

### 3.1. Reagents, Chemicals, and Standards

The reagents included 2,2-difenil-1-picrylidrazyl (DPPH, 98%), butylated hydroxy toluene (BHT, 99%), Folin–Ciocalteu′s phenol reagent (2 M), gallic acid (98%), quercetin (95%), trichloroacetic acid (99%), sodium carbonate (99.5%), aluminum chloride anhydrous (99.99%), potassium ferricyanide (99%), and iron chloride (97%).

The panel of 17 phenolic compounds chosen as reference standards for HPLC-UV-ESI/MS analysis comprised gallic acid (99%), caffeic acid (98%), chlorogenic acid (95%), ferulic acid (99%), rosmarinic acid (98%), catechin (98%), epicatechin (98%), naringin (98%), rutin (94%), luteolin-7-O-glucoside (98%), luteolin (98%), quercetin (95%), apigenin (95%), kaempferol (97%), dihydroquercetin (90%), myricetin (96%), and naringenin (95%).

All chemical reagents and standards were supplied by Sigma Aldrich (Burlington, MA, USA).

The organic solvents (methanol, ethanol, ethyl acetate, and dimethyl sulfoxide) were analytical grade and purchased from local suppliers. Acetonitrile (ACN) (99.9%, Sigma-Aldrich, Saint-Quentin-Fallavier, France) and formic acid (99-100%, AnalaR NORMAPUR^®^, VWR Chemicals, Briare, France) were HPLC grade, and highly purified water was prepared using a Milli-Q water purification system (Millipore, Guyancourt, France).

### 3.2. Plant Material

**Figure 5:**
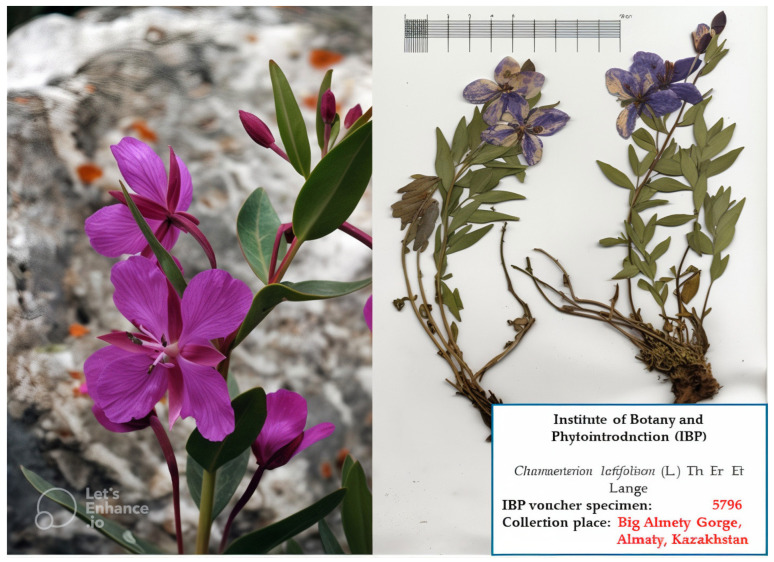
Wild ChL plant (**left**) and the IBP voucher specimen (**right**).

The aerial parts of ChL (leaves and stems) were collected in September 2023 by the Institute of Botany and Phytointroduction (IBP) in the Almaty region (Big Almaty Gorge) of the Republic of Kazakhstan. The plant was identified by the staff at the IBP Herbarium, where the voucher specimen (IBP 5796) has been deposited (Figure 5). After collection, the plant material underwent meticulous cleaning and washing with distilled water. Subsequently, each organ was air-dried in a shaded, well-ventilated area until completely dehydrated, then pulverized into a fine powder using an Electric Micro Plant Grinding Machine (110 V, 1400 rpm/min). All samples were preserved at −20 °C until they were ready for analysis.

### 3.3. Extraction

Twenty grams of dried plant material underwent extraction using a Soxhlet reflux evaporator employing 200 mL of either ethanol or ethyl acetate solvent. Each extraction procedure was conducted over a duration of 5 h. Subsequently, filtrates acquired through this procedure were collected using Whatman filter papers, and the solvents were then removed via evaporation under reduced pressure at 40 °C using a rotary evaporator (IKA RV10 auto V-C, Staufen, Germany). Finally, the semisolid mass underwent transformation into a solidified mass through exposure to ambient air. The quantification of extract contents (W, %) was determined utilizing the following Equation (1), representing the percentage ratio of the final dry mass of extracts (m_1_) to the initial dry mass of the plant material (m_0_) subjected to extraction:

W (%) = (m_1_/m_0_) × 100 (1)

### 3.4. Phytochemical Analysis

#### 3.4.1. Total Phenolic and Flavonoid Content (TPC and TFC)

The total phenolic contents of ethyl acetate and ethanolic extracts of ChL were assessed using the Folin–Ciocalteau method according to Singleton and Rossi with slight modification [75]. First, stock solutions of ChL dry matter (1000 μg/mL) and the standard compound, gallic acid (1000 μg/mL), were prepared in methanol. Then, gallic acid calibration solutions of 25, 50, 75, 100, 150, and 300 μg/mL concentrations were prepared in duplicate. The calibration curve was plotted by mixing of 0.25 mL aliquots of gallic acid standard solutions with 1 mL diluted Folin–Ciocalteau reagent (1:9) and 0.75 mL of 1% sodium carbonate solution. The absorbance was determined at 760 nm (DR3900 Benchtop VIS Spectrophotometer with RFID Technology, Germany), after incubation for 2 h in the dark at room temperature. For both ethyl acetate and ethanolic extracts, 0.25 mL (1000 μg/mL) was mixed separately with the same reagents as performed for the construction of the calibration curve. The concentration of gallic acid in each extract was calculated from the regression equation (y = 0.0073x – 0.0226, R^2^ = 0.99) using its absorbance. Finally, these results were expressed in terms of TPC as milligrams of gallic acid equivalent (GAE) per gram of dry matter (mg GAE/g DM) using the following formula (2):

C = C_1_ × V/m (2)

where C—total phenolic content in mgGAE/g, C_1_—concentration of gallic acid established from the calibration curve in mg/mL, V—volume of the extract in mL, and m—the weight of the dry plant extract in g.

Flavonoid quantification was conducted via direct dosing with aluminum chloride, following the method outlined by Quettier-Delau et al. with minor adjustments [76]. Initially, methanol-based stock solutions of ChL dry matter (1000 μg/mL) and the standard compound, quercetin (1000 μg/mL), were prepared. Subsequently, calibration solutions of quercetin spanning concentrations of 12.5, 25, 50, 75, and 100 μg/mL were prepared in duplicate. The calibration curve was generated by mixing 1 mL of quercetin standard solution with 1 mL of methanolic aluminum chloride (2%). Total flavonoid content (TFC) was determined by combining 1 mL of the extract solution with 1 mL of AlCl_3_ (2%). Following incubation for 15 min at room temperature in darkness, the absorbance readings of all samples were taken at 430 nm using a Hach DR 3900 spectrophotometer. Quercetin concentration in each extract was calculated using the regression equation (y = 0.0691x − 0.0404, R² = 0.99) derived from absorbance values. Finally, TFC values were expressed in milligrams of quercetin equivalent per gram of dry matter (mgQE/g DM).

#### 3.4.2. Analysis by High-Performance Liquid Chromatography with Ultraviolet Detection and Electrospray Ionization Mass Spectrometry (HPLC-UV-ESI/MS)

A high-performance liquid chromatography with ultraviolet detection and electrospray ionization mass spectrometry (HPLC-UV-ESI/MS) system, the Agilent 1260 Infinity (Agilent Technologies, Santa Clara, CA, USA), was employed for the identification and quantification of phenolic compounds in the extracts. The system was equipped with a gradient pump (G1311C 1260 Pump VL), automated sampler (G1329B 1260 ALS), chromatographic oven (G1316A 1260 TCC), variable-wavelength detector (G1314C 1260 VWD VL+), and mass spectrometer (G6130A Quadrupole LC-MS/MS). All processes during HPLC analysis were conducted using NT 4.0 Windows NT-based ChemStation software.

HPLC-UV-ESI/MS conditions: The extracts and standards, dissolved in a solvent mixture of acetonitrile:water (1:1, *v*/*v*), were subjected to chromatographic separations using a “Zorbax Eclipse Plus C18” reversed-phase sorbent column (150 mm × 4.6 mm i.d., 3.5 μm particle size, Agilent Technologies, USA). The protocol involved a gradient elution mode with two solutions: 2.5% (*v*/*v*) formic acid in water (Solution A) and 2.5% (*v*/*v*) formic acid in acetonitrile (Solution B). The gradient program mixed mobile phases A and B in varying proportions, as follows: initially, 3% B in eluent system for the first 5 min, 5.00–10.00 min with 10% B, 10.00–15.00 min with 20% B, 15.00–45.00 min with 30% B, 45.00–50.00 min with 40% B, 50.00–55.00 min with 30% B, and finally with 20% B for 55.00–60.00 min, before returning to 3% B for the last 5 min. The flow rate in the mobile phase was maintained at 0.4 mL/min, and the column temperature was kept constant during the separation process and set at 30 °C. The injection volume for both extracts and standards was 20 µL.

Before entering the mass spectrometry (MS) interface, the column effluent passed through a UV detector, where phenolic compounds were detected at the wavelengths of 280 nm and 360 nm. Mass spectra (ESI+) were recorded within the range of 100–1000 Da. Electrospray ionization mass spectrometry detection, in negative mode, employed optimized parameters: capillary temperature of 350 °C, drying gas N_2_ at 8 L/min, and nebulizer pressure at 45 psi. Data acquisition utilized the multiple reactions monitoring (MRM) method, exclusively monitoring specific mass transitions during predetermined retention times.

The identification of each compound was performed by comparing their retention times with authentic standards and confirmed by an Agilent G6130A LC-MS/MS spectrometer equipped with an electrospray ionization source. The quantitative content of phenolic compounds in the extracts was calculated by the method of an external standard, and the results were expressed in mg/g dry matter [77].

#### 3.4.3. FTIR Analysis

The dried extract samples underwent Fourier transform infrared (FT-IR) spectroscopy analysis using the attenuated total reflectance (ATR) technique employing a diamond cell on a Nicolet^TM^ 6700 instrument (Thermo Scientific, Waltham, MA, USA). Spectra were obtained at a spectral resolution of 4 cm^−1^, with 32 scans conducted per sample within the wavenumber range of 4000–500 cm^−1^. Data acquisition and processing were carried out using Omnic 5.2 software.

### 3.5. Antioxidant Assays

#### 3.5.1. DPPH Radical Scavenging Activity

The capacity of the plant’s ethyl acetate and ethanolic extracts to neutralize radicals was evaluated utilizing the 2,2-diphenyl-1-picrylhydrazyl (DPPH) method, in accordance with a well-defined protocol [78]. Evaluation of DPPH radical scavenging activity was performed by examining six concentrations (0.01, 0.02, 0.04, 0.06, 0.08, and 0.1 mg/mL) of plant extracts or a standard antioxidant dissolved in methanol. Following that, 1 mL of freshly made DPPH solution (0.1 mM in methanol) was mixed with 3 mL of each sample solution, and it was allowed to stand in the dark for 30 min. The absorbance was measured against a blank using a Thermo Scientific Evolution 300 UV-Vis Spectrophotometer (Thermo Electron Scientific Instruments LLC, Madison, WI, USA) after incubating at room temperature. The standard chosen for measuring antioxidant activity was butylated hydroxytoluene (BHT). The entire analysis was performed in triplicate. The following equation (3) was used for calculating the percentage (%) of DPPH radical inhibition:

% inhibition of DPPH = [(A_B_ − A_S_)/A_B_] × 100 (3)

where A_S_ = absorbance of the sample, A_B_ = absorbance of the blank.

The IC_50_ value, which represents the concentration of the sample needed to lower the DPPH radical by 50%, was calculated from a calibration curve by linear regression for each sample and used to express the electron-donating capacity. Lower IC_50_ values correspond to higher antioxidant activities.

#### 3.5.2. FRAP Antioxidant Assay

The ferric reducing capacity of ChL extracts was evaluated by their ability to convert Fe^3+^ to Fe^2+^, following a modified method based on the protocol outlined by Dorman H. et al. [79]. In summary, a solution comprising 2.5 mL of phosphate buffer (0.2 M, pH 6.6) and 2.5 mL of 1% potassium ferricyanide [K_3_Fe(CN)_6_] was prepared, to which varying concentrations of plant extracts or a standard antioxidant (0.01, 0.02, 0.04, 0.06, 0.08, and 0.1 mg/mL) were added (1 mL each). The resultant mixture was then incubated at 50 °C for 20 min, followed by addition of 2.5 mL of 10% trichloroacetic acid and centrifugation at 3000 rpm (Centrifuge 0408, Lab1st, Irvine, CA, USA) for 10 min. Subsequently, 2.5 mL of the supernatant was combined with 2.5 mL of distilled water and 0.5 mL of 0.1% ferric chloride (FeCl_3_), followed by incubation in darkness for 10 min prior to measuring absorbance at 700 nm using a Thermo Scientific Evolution 300 UV-Vis Spectrophotometer (Thermo Electron Scientific Instruments LLC, Madison, WI, USA). Ascorbic acid was used as the standard for this assay. The FRAP antioxidant activity (AOA) was determined according to the following formula:

AOA (%) = ((A_B_ − A_S_)/A_B_) × 100 (4)

where A_S_ = absorbance of the sample, A_B_ = absorbance of the blank.

The experiments were conducted in triplicate, and the results were expressed as mean absorbance values ± standard deviation (SD) and IC_50_ value ± SD.

### 3.6. Antibacterial and Antifungal Activity

The antibacterial and antifungal activities were assessed against a panel of bacterial and fungal strains, including Gram-negative (*Escherichia coli*, *Klebsiella pneumoniae*) and Gram-positive (*Staphylococcus aureus*, *Bacillus cereus*) bacteria, as well as *Candida albicans* fungi. These microbial strains were obtained from the Republican collection of microorganisms (Astana, Kazakhstan). The in vitro antibacterial activity of ChL extracts was evaluated using the disc diffusion method. Whatman filter paper discs impregnated with 90 μL of each extract were prepared according to established procedures and dried until completely dry [80]. Standard discs with a diameter of 5.5 mm, containing either penicillin (10 µg) or nystatin (80 µg) (NICF Ltd., St. Petersburg, Russia, RF), were used as positive controls, while discs infused with 90 µL of sterile deionized water served as negative controls. Nutrient media, such as meat-peptone agar (MPA) and Sabouraud agar, were employed for culturing the reference strains. A suspension of diluted microorganism strains (inoculum) was inoculated onto the agar plates, followed by the placement of extract-infused discs. Incubation of all plates occurred at 37 °C for 18–24 h, after which the inhibition zones around the discs were recorded. Inoculum preparation involved obtaining pure cultures by re-plating isolated colonies onto appropriate agar media. Suspensions of the studied strains were prepared with a McFarland turbidity of 10 IU, resulting in a final concentration of 2 × 10^6^ CFU/mL. Antifungal activity assessment followed a similar protocol, where the *Candida* culture inoculum was plated on sterile Sabouraud agar, and inhibition results were noted accordingly. If the diameter measures less than 1 mm, the strain will be classified as non-sensitive. A diameter ranging between 1.1 and 4.9 mm indicates low sensitivity, while a diameter falling between 5 and 8.9 mm signifies high sensitivity. Strains with a diameter exceeding 9 mm are categorized as extremely sensitive [81].

### 3.7. Statistical Analysis

Statistical analysis of each experimental result was conducted using GraphPad Prism version 5 (GraphPad Software, Inc., La Jolla, CA, USA) for analysis of one-way variance (ANOVA). Duncan’s multiple range test was applied to assess the significance of variations among samples (*p* < 0.05). Data were presented as the mean ± standard deviation derived from triplicate determinations.

## 4. Conclusions

In conclusion, this study highlights the significant potential of ethanol and ethyl acetate extracts from ChL as sources of bioactive compounds with notable antioxidant and antimicrobial properties. The ethanol extract demonstrated higher yields of phenolic and flavonoid compounds, as well as superior antioxidant and antimicrobial activities. Specifically, the ChL-EtOH extract exhibited strong DPPH scavenging activity (IC_50_ = 21.31 ± 0.65 μg/mL) and high reducing capability (IC_50_ = 18.13 ± 0.15 μg/mL), indicating its strong radical scavenging and antioxidant power. The ChL-EtOAc extract also demonstrated considerable antiradical (IC_50_ = 22.60 ± 0.44 μg/mL) and antioxidant (IC_50_ = 21.62 ± 0.22 μg/mL) activities, though it was slightly less effective than the ethanol extract.

These findings underscore the importance of solvent selection in optimizing the extraction of bioactive compounds. Given its superior activities, the ethanol extract holds particular promise for medical and pharmaceutical applications, including as a natural antioxidant and antimicrobial agent. The results provide a scientific basis for the traditional medicinal uses of ChL. Further research should explore the potential medical applications of the ethanol extract, including its use in therapeutic formulations, and investigate the synergistic effects and mechanisms of action of its bioactive compounds to fully harness their therapeutic potential.

## Figures and Tables

**Table 1:** Evaluation of total phenolic and total flavonoid contents of extracts.

Sample	TFC (mg QE/g DM)	TPC (mg GAE/g DM)
ChL-EtOAc	21.72 ± 0.54	42.68 ± 2.48
ChL-EtOH	24.18 ± 1.06	267.48 ± 3.44

QE: Quercetin Equivalent; GAE: Gallic Acid Equivalent; DM: Dry Matter. Results of the ANOVA test are significantly different at *p* < 0.05, and each data point is represented by the average of three repetitions ± SD of one independent experiment.

**Table 2:** Identification and quantification of phenolic compounds in the ethyl acetate and ethanol extracts from the aerial parts of ChL using HPLC-UV-ESI/MS.

Peak No.	Retention Time R_t_ (min)	[M+H]^−^ (m/z)	Identified Metabolites	Subclass	Molecular Formula	Quantity *	
						ChL-EtOH	ChL-EtOAc
1	3.928	179	Caffeic acid	Phenolic acid	C_9_H_8_O_4_	4.40	2.10
2	5.092	169	Gallic acid	Phenolic acid	C_7_H_6_O_5_	35.00	0.80
3	13.466	353	Chlorogenic acid	Phenolic acid (glycoside)	C_16_H_18_O_9_	26.80	3.00
4	17.187	289	Epicatechin	Flavonoid	C_15_H_14_O_6_	-	-
5	19.166	609	Rutin	Flavonoid (glycoside)	C_27_H_30_O_16_	8.80	0.55
6	20.030	463	Quercetin 3-glucoside	Flavonoid (glycoside)	C_21_H_19_O_12_	71.90	2.30
7	20.352	301	Not identified	-	-	-	-
8	20.685	447	Not identified	-	-	-	-
9	20.792	163	p-Coumaric acid	Phenolic acid	C_9_H_8_O_3_	2.70	-
10	22.820	431	Not identified	-	-	-	-
11	23.327	317	Myricetin	Flavonoid	C_15_H_10_O_8_	0.03	0.52
12	30.956	301	Quercetin	Flavonoid	C_15_H_10_O_7_	0.90	0.08
13	33.723	271	Naringenin	Flavonoid	C_15_H_12_O_5_	0.34	0.01
14	38.924	285	Kaempferol	Flavonoid	C_15_H_10_O_6_	0.44	0.82

* Phenolic content in DM (mg/g).

**Table 3:** In vitro antioxidant activity of ChL extracts.

	ChL-EtOAc	ChL-EtOH	BHT	AA
DPPH Test IC_50_ (μg/mL)	22.60 ± 0.44 ^a^	21.31 ± 0.65 ^b^	20.02 ± 0.48 ^c^	ND
FRAP Test IC_50_ (μg/mL)	21.62 ± 0.22 ^a^	18.13 ± 0.15 ^b^	ND	18.64 ± 0.32 ^d^

Values are expressed as the mean ± SD (*n* = 3). Values bearing the same letter (a, b, c, or d) in the same column are significantly different (*p* < 0.05). Butylated hydroxy toluene (BHT) and ascorbic acid (AA) were used as positive controls in antioxidant tests. ND: not determined. IC_50_: Half maximal Inhibitory Concentration.

**Table 4:** Antibacterial and antifungal activities of ChL extracts.

Microorganisms Tested	Gram Type	Extract		Positive Control	
		ChL-EtOH, IZD, mm	ChL-EtOAc, IZD, mm	Penicillin, IZD, mm	Nystatin, IZD, mm
*Escherichia coli*	Gram −	8.53 ± 0.12	NA	NA	-
*Klebsiella pneumonia*	Gram −	9.26 ± 0.08	NA	NA	-
*Staphylococcus aureus*	Gram +	11.06 ± 0.30	NA	30.26 ± 0.88	-
*Bacillus cereus*	Gram +	9.60 ± 0.24	NA	18.63 ± 0.32	-
*Candida albicans*	Fungus	14.27 ± 0.65	8.58 ± 0.22	-	11.28 ± 0.28

IZD: Inhibition Zone Diameter. NA: no activity. Results presented as mean ± SD. Note: The antagonistic activity of the studied cultures is considered zero when the width of the zone of no growth is up to 1.0 mm, low—at 1.1–4.9 mm, medium—at 5.0–8.9 mm, and high—at 9.0 mm or more.

## Data Availability

Data are contained within the article and Supplementary Material.

## Supplementary Materials

The following supporting information can be downloaded at: https://www.mdpi.com/article/10.3390/ph17080996/s1, Figure S1: Antimicrobial activity of ChL extracts determined by the disk diffusion method; Table S1: FT-IR peak values and associated functional groups identified in the spectra of ethyl acetate and ethanol extracts of *C. latifolium*.
