# Supplementary material for: Phytochemical Profiling, Antioxidant and Antimicrobial Potentials of Ethanol and Ethyl Acetate Extracts of Chamaenerion latifolium L
Source: Pharmaceuticals (Basel). 2024 Jul 27;17(8):996. doi: 10.3390/ph17080996 (PMC11357188; doi:10.3390/ph17080996)
Supplement: Supplementary file 1 [file pharmaceuticals-17-00996-s001.zip › pharmaceuticals-3101832-supplementary.pdf]

Supplementary Data

Table S1. FT-IR peak values and associated functional groups identified in the spectra of ethyl acetate and ethanol extracts of *C. latifolium*.

Wavenumbers (cm^{-1})		Functional Groups	Bond
**ChL-EtOAc**	**ChL-EtOH**		
3297.63	3258.30	Alcohol/ Phenol	O-H stretching
2917.79	2933.93	Alkane/ Alcohol (acid)	C-H stretching O-H stretching
2849.58	2850.64		
1709.34	1709.21	Carbonyl	C=O stretching
1691.29	-	Conjugated acid	C=O stretching
1653.39	1653.46	Conjugated alkene	C=C stretching
1605.66	1605.67	Cyclic alkene, Aromatic compound	C=C stretching
1506.34	1506.52	Aromatic compound	C=C stretching
1446.71	1446.68	Alcohol/ Carboxylic acid	O-H bending
1357.06	-		
1197.34	1197.25	Alkyl/aryl ether	C-O stretching
1171.25	-	Alcohol tertiary/ Aromatic compound	C-O stretching/ C-H in-plane bending
1030.89	1027.72	Sulfoxide/ Aromatic compound	S=O stretching/ C-H in-plane bending
-	864.13	Alkane (1,2,4-trisubstituted)/ Aromatic compound	C-H bending/ C-H out-of-plane bending
-	817.37	Alkene/ Aromatic compound	C=C bending/ C-H out-of-plane bending
811.45	-		
717.86	-		

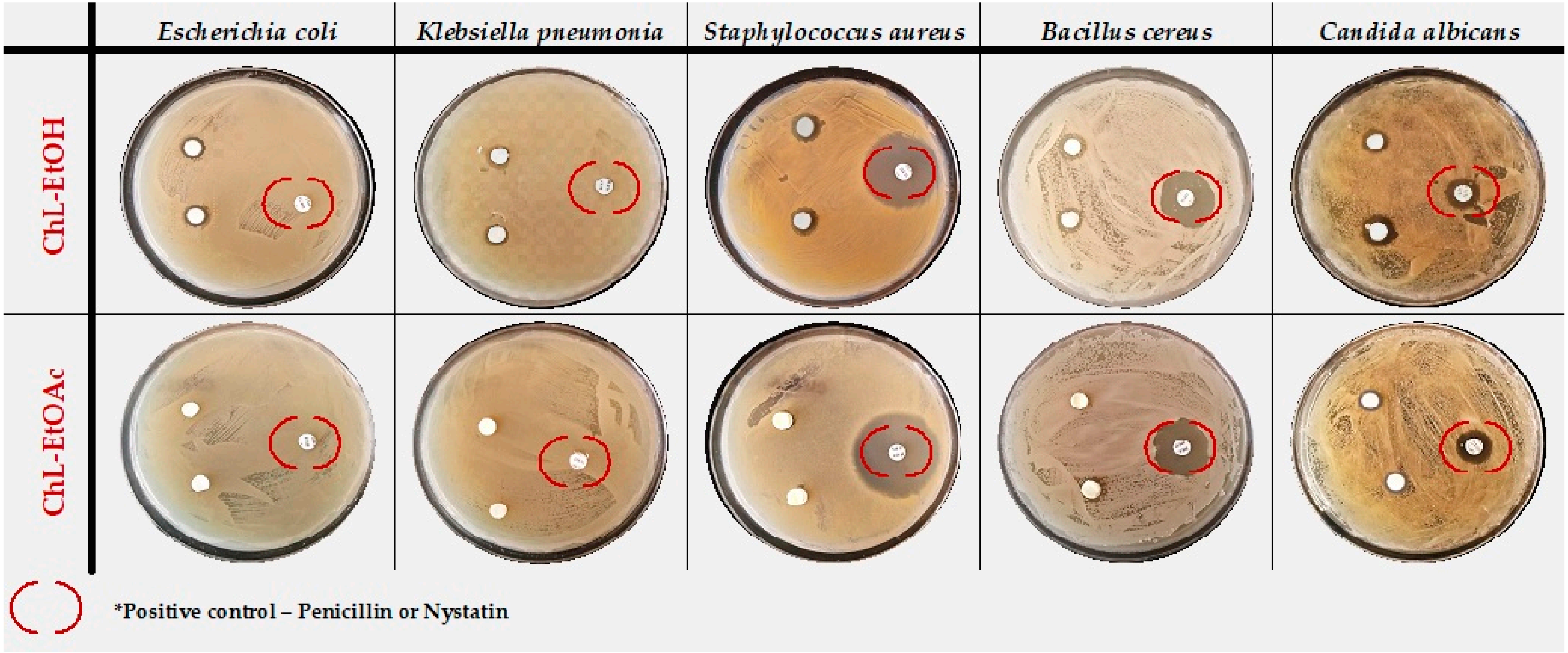

Figure S1. Antimicrobial activity of ChL extracts determined by the disk diffusion method
